# Intratumoral genetic heterogeneity in metastatic melanoma is accompanied by variation in malignant behaviors

**DOI:** 10.1186/1755-8794-6-40

**Published:** 2013-10-11

**Authors:** Matthew Anaka, Christopher Hudson, Pu-Han Lo, Hongdo Do, Otavia L Caballero, Ian D Davis, Alexander Dobrovic, Jonathan Cebon, Andreas Behren

**Affiliations:** 1 Cancer Immuno-biology Lab, Ludwig Institute for Cancer Research Melbourne, Austin Branch, Melbourne, Victoria 3084, Australia; 2 Department of Medicine, Austin Health, University of Melbourne, Parkville, Victoria 3010, Australia; 3 Molecular Pathology Research and Development Laboratory, Peter MacCallum Cancer Centre, Melbourne, Victoria 3002, Australia; 4 Department of Pathology, University of Melbourne, Parkville, Victoria 3010, Australia; 5 Department of Neurosurgery, John Hopkins University School of Medicine, Baltimore, MD 21231, USA; 6 Uro-Oncology Lab, Ludwig Institute for Cancer Research Melbourne, Austin Branch, Melbourne, Victoria 3084, Australia; 7 Current Address: Monash University and Eastern Health, Box Hill, Victoria 3128, Australia; 8 Ludwig Institute For Cancer Research, Level 5, Olivia Newton-John Cancer & Wellness Centre, Studley Road, Heidelberg, Victoria 3084, Australia

**Keywords:** Melanoma, Microarray, Heterogeneity, Mutation profiling, Copy number, Clonal

## Abstract

**Background:**

Intratumoral heterogeneity is a major obstacle for the treatment of cancer, as the presence of even minor populations that are insensitive to therapy can lead to disease relapse. Increased clonal diversity has been correlated with a poor prognosis for cancer patients, and we therefore examined genetic, transcriptional, and functional diversity in metastatic melanoma.

**Methods:**

Amplicon sequencing and SNP microarrays were used to profile somatic mutations and DNA copy number changes in multiple regions from metastatic lesions. Clonal genetic and transcriptional heterogeneity was also assessed in single cell clones from early passage cell lines, which were then subjected to clonogenicity and drug sensitivity assays.

**Results:**

MAPK pathway and tumor suppressor mutations were identified in all regions of the melanoma metastases analyzed. In contrast, we identified copy number abnormalities present in only some regions in addition to homogeneously present changes, suggesting ongoing genetic evolution following metastatic spread. Copy number heterogeneity from a tumor was represented in matched cell line clones, which also varied in their clonogenicity and drug sensitivity. Minor clones were identified based on dissimilarity to the parental cell line, and these clones were the most clonogenic and least sensitive to drugs. Finally, treatment of a polyclonal cell line with paclitaxel to enrich for drug-resistant cells resulted in the adoption of a gene expression profile with features of one of the minor clones, supporting the idea that these populations can mediate disease relapse.

**Conclusion:**

Our results support the hypothesis that minor clones might have major consequences for patient outcomes in melanoma.

## Background

Cancer is caused by successive genetic change that disrupts regulatory processes and endows cells with survival and growth advantages [1]. Ongoing mutation provides a substrate on which selection operates, with aberrations yielding increased fitness leading to an increasing proportion of affected cells and their progeny [2]. Clonal genetic diversity of cancer cells has been correlated with poor prognosis for cancer patients [3]. In pancreatic [4] and renal cancer [5], exome sequencing of different regions of primary and metastatic tumors has identified heterogeneity in sequence mutations. These findings are of particular interest given the current focus in oncology on using drugs that target specific mutant proteins and downstream signaling nodes.

Melanomas can contain tens of thousands of mutations [6,7]. While metastases can be genetically divergent from primary tumors [8,9], heterogeneous *BRAF* mutation status has also been demonstrated between individual circulating melanoma cells [10]. In primary and metastatic lesions, Takata et al. [9] demonstrated different clonal heterogeneity using microsatellite markers mapping to chromosomes 6q, 9p, 10q and 18q to assess LOH. Recently, a heterogeneously present *NRAS* mutation was reported in a progressing lesion following treatment with vemurafenib [11]. However, there has been no genome wide characterization of genetic heterogeneity within metastatic melanoma lesions to date. Likewise it is unknown whether cell lines retain genetic heterogeneity representative of the original tumor.

In this study we assessed genetic heterogeneity in metastatic melanomas and derived cell lines at the level of copy number abnormalities and sequence mutations in a cancer-focused panel of genes. We found significant copy number heterogeneity in tumors and cell lines, and went on to demonstrate that much of the functional heterogeneity we observed could be attributed to relatively minor clones.

## Results

### Regional DNA copy number heterogeneity in metastatic melanoma

**Figure 1:**
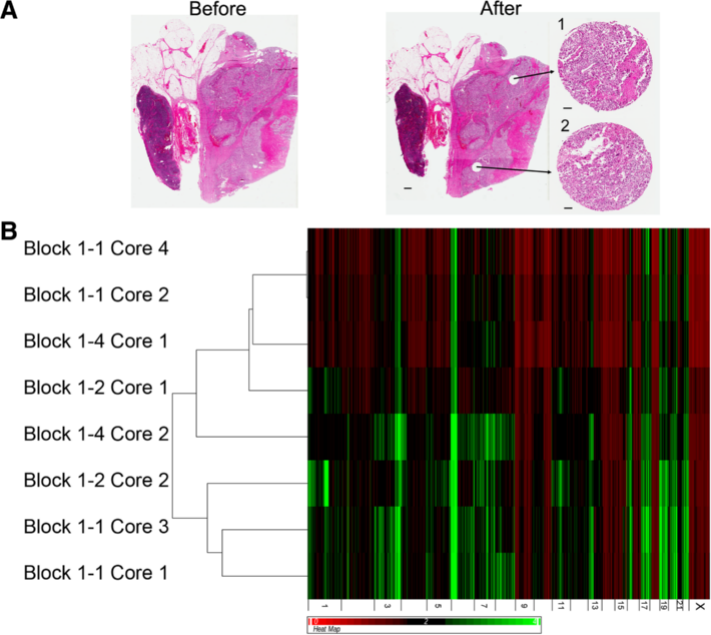
**Copy number heterogeneity between different regions of a metastatic melanoma tissue sample. A)** Representative H&E staining of section of FFPE block from Tumor 1 before coring and after coring. Inserts in the ‘after’ panel are H&E stains from the bottom of the core fragment used for DNA isolation. Scale bar next to whole section represents 1 mm, bars next to cores represent 100 μm. **B)** Hierarchical clustering and heatmap of copy number data from Tumor 1 cores. Black regions represent normal copy number (2), green represents amplifications, red deletions.

**Figure 2:**
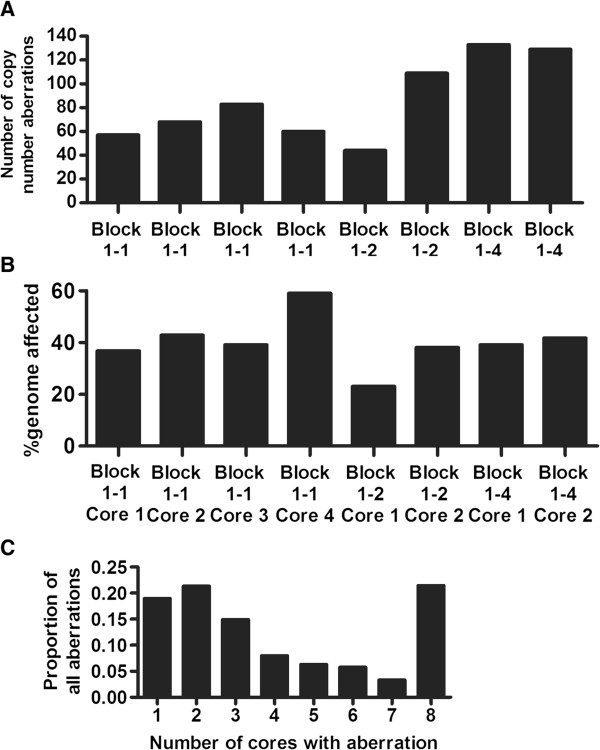
**Attributes of copy number variation in different regions of Tumor 1. A)** Total number of copy number abnormalities identified in each core. **B)** Percent of genome affected by copy number abnormalities identified in each core. **C)** Proportion of cores in which the copy number abnormalities were identified.

**Figure 3:**
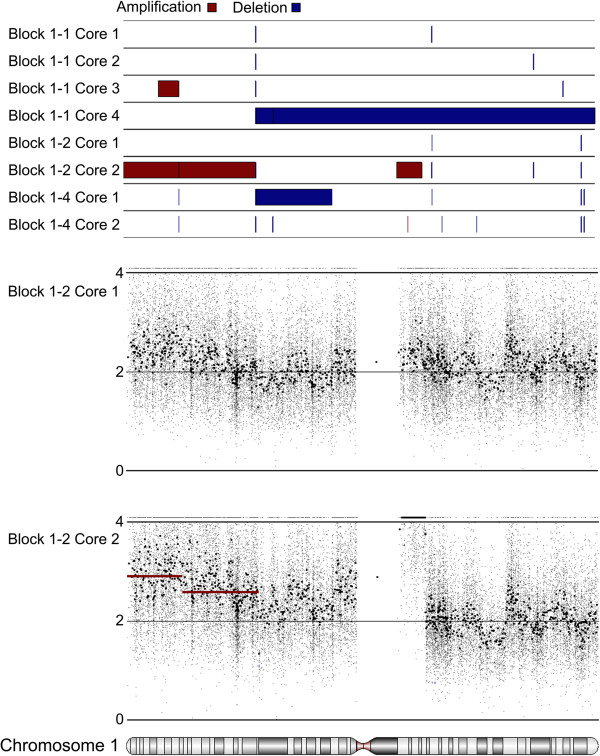
**Copy number heterogeneity of Chromosome 1 in different regions of Tumor 1.** Segmentation results for all eight cores are shown in the top panel; amplification in red, deletions in blue. The plots below show results for Block 1–2 Core 1 and Block 1–2 Core 2 in greater detail. Regions defined by segmentation are highlighted by solid red and blue bars as above. Small dots represent the copy number values of individual array probes, while larger dots represent smoothed data resulting from averaging results from 30 adjacent probes. The high level amplification of 1q21, encompassing SETDB1, can clearly be seen adjacent to the centromere in Core 2 of Block 1–2.

Eight regions of lymph node metastasis Tumor 1 were assessed for the presence of chromosomal amplifications and deletions. DNA extracted from cores taken from three separate FFPE tissue blocks was analyzed using the Affymetrix Oncoscan 2.0 platform. H&E staining was used to identify regions composed primarily of tumor cells prior to coring, with sections taken from immediately below analyzed fragments to control for contaminating normal tissue (Figure 1A and Additional file 1: Figure S1). Hierarchical clustering of DNA copy number profiles separated the samples into two groups, with visual inspection of the heatmap showing that cores taken from the same tissue block often demonstrated very different patterns of amplifications and deletions (Figure 1B). Statistically significant regions of amplification and deletion were next defined using a segmentation algorithm, and the occurrence of specific aberrations compared across the tumor regions. The sampled tumor regions harbored between 44 and 133 significant regions of copy number changes (Figure 2A), encompassing between 23 and 59 percent of the genome (Figure 2B). The greatest proportion of changes was present in all regions; however, many aberrations were present in only one or two cores (Figure 2C). Heterogeneity was observed in genomic regions containing genes with demonstrated potential to impact melanoma biology, such as the high level amplification (greater than 5 copies) of chromosome band 1q21 observed in Core 2 from Block 1–2. This region encompasses the gene for histone methyltransferase SETDB1, recently identified as an oncogene [12] and a candidate susceptibility gene [13] in melanoma. Detailed probe level and segmentation results from Chromosome 1 and Chromosome 17 are shown in Figure 3 and Additional file 2: Figure S2 respectively.

The Oncoscan 2.0 platform also includes probes that test for 541 individual mutations in 62 well known cancer genes, with the presence of a mutation indicated by high probe intensity. Despite the presence of mutation-specific probes with highly heterogeneous intensity, no heterogeneous mutations could be validated by capillary sequencing. Similar results were obtained following Ion Torrent sequencing of amplicons covering a similar panel of cancer genes, which was performed in regions from two additional melanomas (Tumors 2 and 3). These experiments are described in the Additional file 3, in Additional file 4: Table S1, and in Additional file 5: Figures S3 and Additional file 6: Figure S4.

### Early passage metastatic melanoma cell lines retain genetic heterogeneity

**Figure 4:**
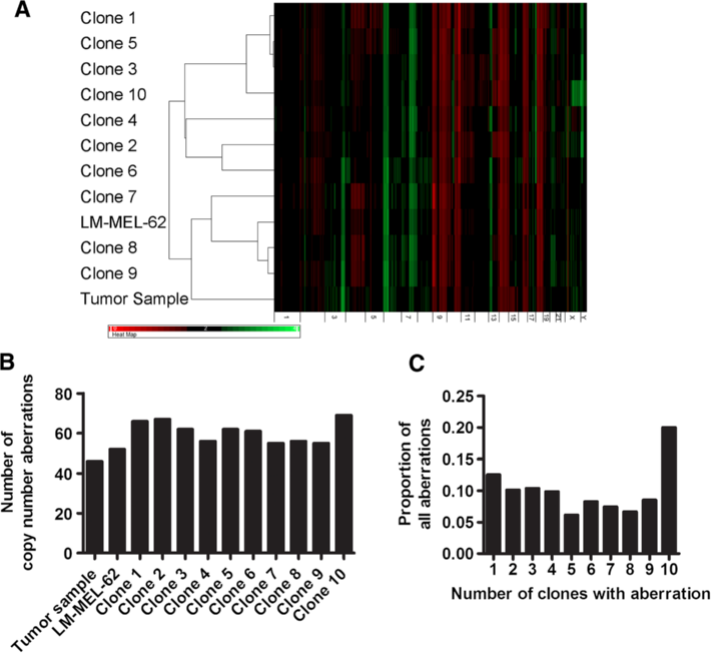
**LM-MEL-62 features clonal copy number heterogeneity. A)** Hierarchical clustering and heatmap of copy number data from single cell clones of LM-MEL-62, the parental cell line, and a fresh frozen fragment of Tumor 1. Black regions represent normal copy number (2), green represents amplifications, red deletions. **B)** number of copy number abnormalities found in from single cell clones of LM-MEL-62, the parental cell line, and a fresh frozen fragment of Tumor 1. **C)** Proportion of clones in which specific copy number changes were identified.

**Figure 5:**
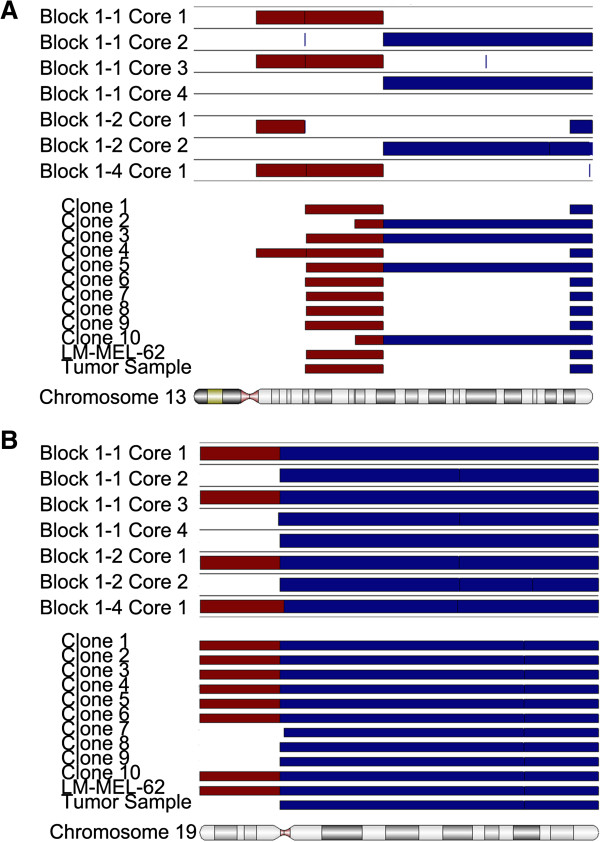
**In vitro clonal copy number heterogeneity can recapitulate heterogeneity found in the original tumor.** Comparison of amplifications, deletions, and break points identified on Chromosome 13 **(A)** and Chromosome 19 **(B)** in FFPE cores from multiple regions of Tumor 1, and matched cell line LM-MEL-62, derived single cell clones, and independent fresh frozen fragment of Tumor 1.

The copy number profiles of a cell line derived from Tumor 1 (LM-MEL-62), ten single cell clones derived from LM-MEL-62, and a fresh frozen tumor fragment of Tumor 1 were assessed using Illumina SNP microarrays. All cell line-derived samples clearly shared many common copy number changes with the original tumor, such as gain of 6p (Figure 4A). The clones harbored between 55 and 69 copy number changes, which represents significantly less variation than was observed in the different regions of Tumor 1 (Figure 4B). Nevertheless, the cell clones were also heterogeneous for many of the detected aberrations (Figure 4C), and the LM-MEL-62 clones displayed heterogeneity at chromosome regions similarly affected in the archival FFPE tumor material (Figure 5A and B). This suggests that early passage melanoma cell lines are polyclonal, and that they can retain or recapitulate genetic heterogeneity representative of that found in the patient’s tumor.

**Figure 6:**
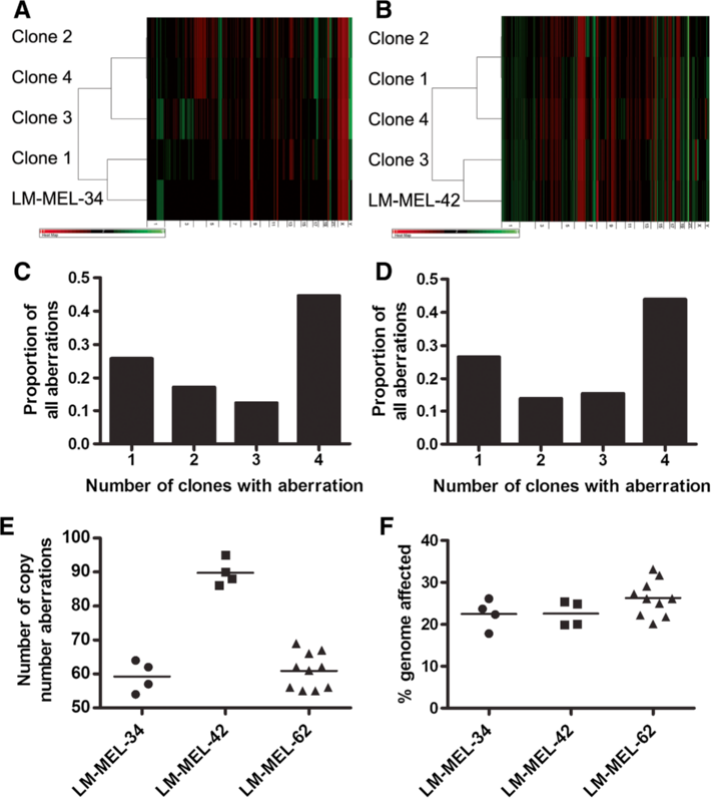
**Copy number heterogeneity in single cell clones from metastatic melanoma cell lines LM-MEL-34 and LM-MEL-42.** Hierarchical clustering and heatmap of copy number data from single cell clones of LM-MEL-34 **(A)** and LM-MEL-42 **(B)** along with the parental cell lines. LM-MEL-34 was established from Tumor 2, while LM-MEL-42 was established from Tumor 3. Black regions represent normal copy number (2), green represents amplifications, red deletions. **C)** Proportion of clones from LM-MEL-34 in which specific copy number changes were identified. **D)** Proportion of clones from LM-MEL-42 in which specific copy number changes were identified. **E)** Total number of copy number abnormalities found in clones from three metastatic melanoma cell lines. **F)** Percent genome affected by copy number abnormalities found in clones from three metastatic melanoma cell lines.

We generated copy number profiles for clones from two other cell lines derived from Tumor 2 and Tumor 3 (Figure 6A & B respectively). Clones from both cell lines had copy number changes that were identified in all clones, and some that were present in some clones but not others (Figure 6C & D). The clones from the three cell lines assessed contained significantly different quantities of copy number changes (one way ANOVA, p < 0.0001; Figure 6E), however they did not differ in the proportion of the genome affected by copy number abnormalities (one way ANOVA, p = 0.165; Figure 6F).

The copy number profile of the parental cell lines represent an average signal derived from all cells in the line. Therefore, the clones with profile closest to the parental line as determined by clustering (such as Clones 7, 8, and 9 in LM-MEL-62) represent the most prevalent or dominant clone(s), with the others representing relatively minor populations. This information provided the opportunity to assess functional differences between dominant and minor cell populations.

### Single cell clones from a metastatic melanoma cell line are functionally heterogeneous

**Figure 7:**
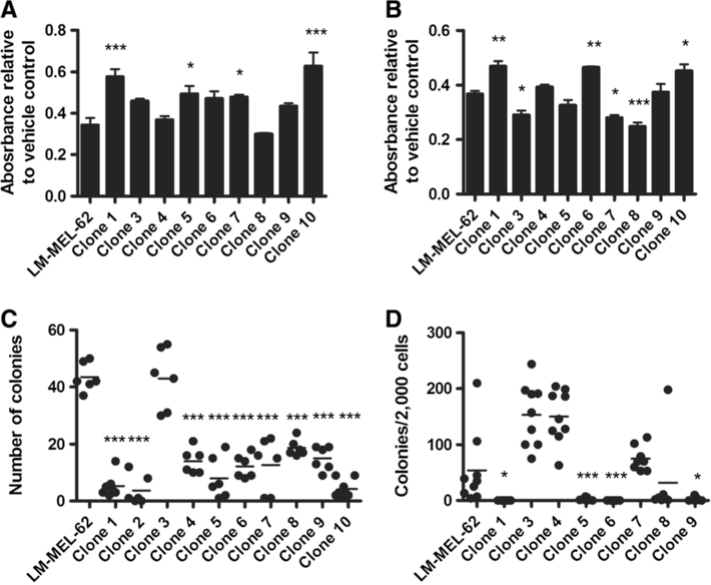
**Functional heterogeneity between clones of LM-MEL-62.** Sensitivity of LM-MEL-62 and derived clones to 20nM paclitaxel **(A)** and 200μM 5FU **(B)**. Values plotted represent a ratio of the MTS absorbance of drug treated samples to vehicle treated controls. Data from Clones were compared to parental LM-MEL-62 by one-way ANOVA with Dunnett’s Multiple Comparison Test. P-value < 0.05 – *; < 0.001 – ***. **C)** Single cell colony formation assay to measure growth independence of LM-MEL-62 and derived clones. Clones were compared to parental LM-MEL-62 using a one-way ANOVA with Dunnett’s Multiple Comparison Test. P-value < 0.001 – ***. **D)** Soft-agar colony formation assays to measure anchorage independent growth and clonogenicity of LM-MEL-62 and derived clones. Clones were compared to parental LM-MEL-62 using a one-way ANOVA with Dunnett’s Multiple Comparison Test. P-value < 0.05 – *; < 0.001 – ***.

In order to assess whether the genetic heterogeneity observed in cell lines was accompanied by functional variation, we compared clones from LM-MEL-62 to the parental line. Clear differences were seen in their sensitivity to cytotoxic drugs paclitaxel (Figure 7A) and 5-fluorouracil (5FU) (Figure 7B), and in their ability to form adherent colonies from single cells (Figure 7C), and in soft-agar (Figure 7D). Clones with copy number profiles that did not cluster with the parental line (presumably less prevalent in the pooled population) were those that demonstrated the strongest behaviors. For example, Clone 3 was the most clonogenic, and Clones 1 and 10 were the least sensitive to cytotoxic drugs. Clone 2 was not assessed for drug sensitivity, and Clone 10 was not assessed in soft-agar assays, as they ceased proliferating before the assays could be performed. This indicates that the clones also differed in their long-term replicative potential following isolation.

### Single cell clones from metastatic melanoma cell lines display evidence of differential pathway activation based on gene expression profiling

**Figure 8:**
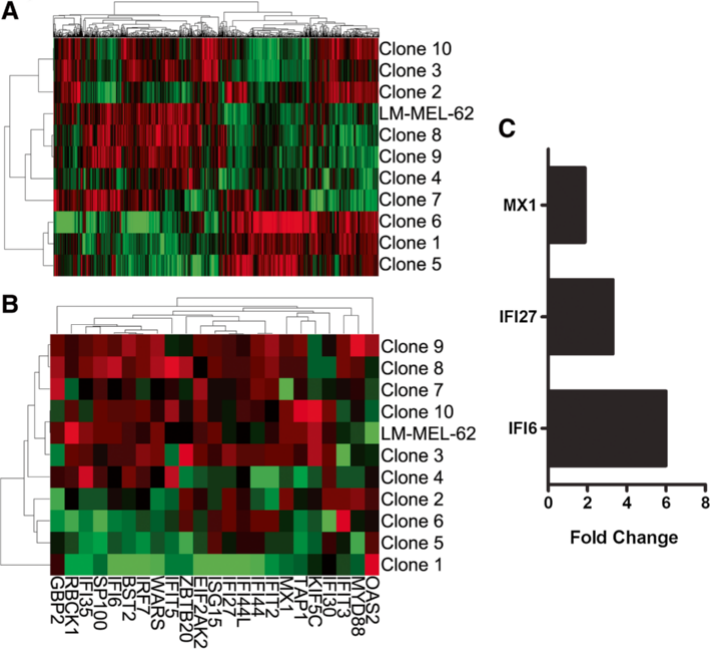
**Gene expression heterogeneity in single cell clones of metastatic melanoma cell line LM-MEL-62. A)** Hierarchical clustering based on the 1000 genes with the greatest variants amongst all samples. **B)** Hierarchical clustering of the LM-MEL-62 and derived clones using genes from MSigDB gene set M9221 with positive enrichment in Clones 1, 5, & 6 (Table 2). **C)** Expression of interferon-inducible genes was significantly increased in a paclitaxel-resistant LM-MEL-62 derivative relative to the parental cell line (P = 0.0012; paired t-test) based on QPCR analysis.

The gene expression profiles of LM-MEL-62 and derived clones were assessed using Illumina HT-12 microarrays. Unsupervised hierarchical clustering placed some clones into different clusters than observed based on copy number data, but Clones 7, 8, and 9 still clustered with the parental cell line (Figure 8A). Again as the profile of the parental line represents an average of all cells/clones, this supports Clones 7, 8, & 9 as the most prevalent cell types.

The clones segregated into three clusters, which we compared using gene set enrichment analysis (GSEA) (Table 1, full results in Additional file 7: Table S2). Interestingly, the clones with the expression profile most similar to the parental cell line expressed genes typical of aggressive metastatic melanoma. Their expression profile was similar to one derived by comparing melanoma metastasis to primaries, and from the primaries of patients that later developed metastases. In contrast, the other groups of clones over-expressed genes related to the activity of specific signaling pathways, such as MET and PI3K, and genes induced by interferon-alpha.

**Table 1:** Selected gene sets associated with clustering-defined groups of clones of melanoma cell line LM-MEL-62

**Gene set description**	**NES**	**FDR**	**MSigDB ID**
**Genes sets enriched in Clones 4,7,8,9**			
Up-regulated in melanoma patients with distant metastasis within 4 years	2.60	0.000	M6387
DNA repair and replication genes up-regulated in melanoma patients who relapsed	2.46	0.000	M2431
Up-regulated in metastases to primary melanomas	2.14	0.000	M5740
**Genes sets enriched in Clones 1,5,6**			
Genes up-regulated in fibroblasts after interferon alpha treatment	2.10	0.000	M9221
Tumor necrosis factor pathway	1.95	0.003	M18030
NOTCH signaling	1.89	0.006	M7946
**Genes sets enriched in 2,3,10**			
Genes up-regulated in MET over-expressing colon cancer xenografts	2.23	0.000	M3231
Genes involved in G beta:gamma signaling through PI3Kgamma	1.79	0.046	M14301

NES – normalized enrichment score. FDR – false discovery rate.

Notably, the expression of interferon-inducible genes has been associated with decreased sensitivity to paclitaxel [14,15]. Consistent with this, the clone expressing the highest levels of inflammation and interferon-inducible genes (Clone 1; Figure 8B) was significantly less sensitive to paclitaxel than the parental cell line (Figure 7A). Treating parental LM-MEL-62 with increasing concentrations of paclitaxel likewise enriched for a cell population that expressed higher levels of several of interferon inducible genes from the enriched gene sets (Figure 8C). This demonstrates that a phenotype that was present in only a minority of cells has the potential to play an important role in survival and re-growth during and after drug treatment.

## Discussion

Excision of localized primary melanomas provides a curative treatment for most patients; however, survival for patients with disseminated disease treated with systematic therapies remains poor. Clonal diversity has been associated with poor prognosis and treatment resistance in cancer [3], and we therefore examined genetic diversity in melanoma metastases.

We found that presumed disease drivers, such as MAPK activating mutations, were homogeneously present in metastatic lesions. In contrast we identified significant heterogeneity in chromosomal aberrations in different regions of a lymph node metastasis, and in clones from several cell lines, suggesting that genetic evolution continues in metastases. The clones from LM-MEL-62 contained chromosomal heterogeneity at regions similar to those in the original tumor, and were additionally heterogeneous in their gene expression profile and cellular behaviors.

This intratumoral heterogeneity becomes significant if it provides populations of cells capable of surviving and proliferating following changes in selective pressures, such as during drug treatments. As a model of this possibility, we selected for a population of cells resistant to paclitaxel, and observed the outgrowth of cells with an interferon-inducible gene expression signature originally found in only a minor population of paclitaxel-insensitive cells in the parental cell line. A recent study in which clonal populations of colorectal cancer cells were tracked in xenografts likewise found that clones that were initially rare went on to become the dominant cell population following treatment with chemotherapy [16]. Likewise our results complement a recent report stating that based on the number of cells in established tumors and a conservative mutation rate, clones containing mutations conferring drug resistance will inevitably be present pre-treatment [17]. In our study other minor clones expressed genes related to the PI3K and MET pathways, which have been associated with resistance to vemurafenib [18,19]. The presence of minor clones with higher baseline activity of these pathways could contribute to targeted therapy resistance, much as we observed with paclitaxel treatment.

Finally it is important to note that despite using two independent technologies to profile somatic mutations, both of which were designed for use with DNA from formalin fixed tissues, we encountered many false positives. Although this issue might be avoided by using frozen tissue, formalin fixation will remain a favored way to preserve samples due to ease of storage and other advantages. We therefore urge caution when using these samples with platforms like the Ion Ampliseq panels, as fixation-related artifacts may will likely lead to false positive variants calls.

## Conclusion

Metastatic melanomas feature significant genetic and phenotypic heterogeneity, with the potential to confound the success of many therapies. Genetic heterogeneity represents an obstacle for mutation-directed personalized cancer medicine, albeit one which might be compensated for by analyzing multiple biopsies at sufficient depth to identify rare variants. However, drug resistance can have its basis in genetic, epigenetic, and stochastic variation, and the transcriptomic and functional variation we identified in this study suggests that these mechanisms are relevant in metastatic melanoma. As accounting for these additional levels of regulation in multiple biopsies would be exceedingly challenging, targeting multiple tumor clones might best be accomplished using immunotherapy approaches such as tumor-lysate stimulated adoptive cell transfer or immune regulatory check point blockade agents such as ipilimumab. These therapies have intrinsic potential to induce responses against a broad range of antigens specific to a patient’s tumor, which would circumvent the type of sampling error caused by intratumor heterogeneity that must be confronted in attempting to choose appropriate drugs based on the analysis of biopsies.

## Methods

### Tumor material and cell lines

Melanoma lymph node metastases and derived early passage cells lines from three patients were analyzed for regional and clonal differences in this study. Following pathological examination tumors were used for cell line generation, archived as formalin fixed paraffin embedded (FFPE) tissue blocks, and nucleic acid extraction performed from snap frozen fragments when sufficient material was available. Tissue donors consented for tissue collection and protocols were approved by the Austin Health Human Research Ethics Committee, Melbourne, Australia. The clinical characteristics of all samples are listed in the Table 2. In addition to a splenic lymph node lesion, the patient associated with Tumor 3 had a hepatic artery lymph node deposit resected concurrently, which was labeled as Tumor 3 Block 2–4.

**Table 2:** Tumor and cell line characteristics

	**Site of disease**	**Stage at resection**	**Sex**	**Derived cell line**
**Tumor 1**	Axillary lymph node	IIIc	Male	LM-MEL-62
**Tumor 2**	Axillary lymph node	IIIb/c	Female	LM-MEL-34
**Tumor 3**	Splenic lymph node	IVc	Male	LM-MEL-42

The FFPE blocks from an individual tumor represent contiguous transverse slices; however, tissue orientation was not recorded during embedding. Hematoxylin and eosin (H&E) staining was used to determine regions of viable tumor cells in each tissue block, and 3mm cores were then removed from target regions using a tissue microarrayer. The tip (~2 mm) of the cylindrical core was removed with a sterile scalpel blade and used for DNA extraction. For the cores from Tumor 1 used for DNA copy number analysis, the remaining core was embedded into a recipient block of paraffin so that the upper surface could be sectioned and the proportion of tumor cells analysed. Cores from Tumors 1 and 2 were used only for sequencing-based mutation profiling.

The melanoma cell line establishment, culture methods, and RF10 growth media formulation used by our laboratory have previously been reported [20]. Single cell-derived clonal sublines (clones) were isolated through low-density plating and colony isolation using 5 mm plastic cylinders.

### Microarray analysis

Nucleic acids were extracted from cell line pellets and fresh frozen tumor pieces using the AllPrep Mini Kit (Qiagen). All extractions for cell line clones were performed before the clones had been passaged five times in culture. DNA extraction from FFPE samples used the Arcturus Picopure DNA Extraction kit (Applied Biosystems) with a 24 hour Proteinase K incubation, followed by further purification using DNeasy columns (Qiagen).

DNA from cell lines, patient blood, and fresh frozen materials was analyzed on Illumina Human610-Quad genotyping arrays at the Memorial Sloan-Kettering Cancer Center Genomics Core Laboratory and imported into Partek Genomics Suite (PGS). Data from blood samples was used to create paired copy number data for each patient. Segmentation algorithm settings were: minimum markers 15, p-value 0.0001, expected range 0.5, amplification signal-to-noise ratio 0.4, deletion signal-to-noise ratio 0.8.

DNA from FFPE samples was analysed using the Oncoscan Express 2.0 service from Affymetrix, which employs arrays containing 334 000 copy number probes, and 541 probes specific for somatic cancer mutations. Oncoscan copy number data were processed and normalized by Affymetrix according to previously published methods [21], and copy number is calculated in reference to Oncoscan data from an Affymetrix panel of normal reference samples. Segmentation algorithm settings were: minimum markers 10, p-value 0.0001, expected range 0.5, amplification signal-to-noise ratio 0.4, deletion signal-to-noise ratio 1.0.

Hierarchical clustering of copy number data used Euclidian distance and average linkage.

Illumina HT-12 gene expression arrays were processed at the Australian Genome Research Facility. Using R and the Bioconductor package Limma [22], raw data were background corrected using the *normexp* function, log-transformed, and quantile normalized. Hierarchical clustering in Partek GS used Euclidian distance and average linkage. Gene Set Enrichment Analysis (GSEA) [23] employed gene set permutation, a 5% FDR cutoff, and gene sets from the MSigDB database v3.0, category C2.

The Illumina HT-12 gene expression array and Illumina 610-Quad SNP array data are available in the ArrayExpress database (http://www.ebi.ac.uk/arrayexpress) under accession number E-MTAB-1753.

### Mutation profiling by amplicon sequencing

Somatic mutation profiling using the Ion AmpliSeq Cancer Panel, Ion Torrent sequencing, and the Ion Variant Caller (Life Technologies) was performed by AIT Biotech (Singapore). Libraries were barcoded using the Ion Xpress Barcode Kit and eight samples were sequencing together in a single Ion Torrent sequencing run using Ion 318 chips (all Life Technologies). Default Ion Variant Caller settings were employed, with the exception of a SNP QV minimum of 14. This value was chosen as it eliminated false negatives in the identification of *BRAF* mutations in Tumor 1, which was previously confirmed via capillary sequencing. Known SNPs and synonymous changes were removed, as were low-confidence insertions and deletions associated with homopolymer runs were also disregarded due to known issues with false positives using this sequencing platform [24]. Variants were analyzed using the Ensemble Variant Effect Predictor [25], which included SIFT [26] and PolyPhen [27] predictions of the consequence of a sequence variant on protein function. The amplicon sequencing reads and variant prediction results are available in the ArrayExpress database (http://www.ebi.ac.uk/arrayexpress) under accession number E-MTAB-1794.

Validation of high frequency variants was performed using standard capillary sequencing. PCR products covering mutations indentified by Ion Torrent sequencing were amplified using GoTaq mastermix (Promega). PCR products were sequenced at AGRF, using a 3730xl sequencer and BigDye Terminator v3.1 chemistry (both Applied Biosystems). Primers (all shown 5′ to 3′) used to validate the BRAF and NRAS mutations in Tumor 1 were as follows: BRAF G649E Forward Primer – TACCATGCCACTTTCCCTTG, Reverse Primer TTTTCTGTTTGGCTTGACTTGA; NRAS G12S Forward Primer – GGTTTCCAACAGGTTCTTGC, Reverse Primer CTCACCTCTATGGTGGGATCA.

Validation for low frequency variants was performed by high-resolution melt (HRM) analysis on a Rotor-Gene Q (Qiagen), before and after uracil-DNA glycosylase (UDG) treatment to control for FFPE sequence artifacts as previously described [28]. The HRM primer sequences for FGFR3 exon 6 variant analysis were F 5′-CAGTGGCGGTGGTGGTGAGG-3′ and R 5′-ACCTTGCAGTGGAACTCCACGTC-3′. PCR cycling and HRM was performed on the Rotor-Gene Q (Qiagen, Hilden, Germany). The reaction mixture in a final volume of 20 μL was prepared as follows; 1 × PCR buffer, 2.5 mM MgCl2, 400 nM of each primer, 10 ng of FFPE DNA, 200 μM of dNTPs, 5 μM of SYTO9 (Invitrogen), and 0.5 U of HotStarTaq polymerase (Qiagen). The PCR cycling and melting conditions were as follows; an initial incubation at 95°C for 15 min, followed by 55 cycles of 96°C for 15 s, 70°C for 20 s, 72°C for 30 s; one cycle of 97°C for 1 min and a melt from 70°C to 95°C rising 0.2°C per second. All samples were tested in duplicate. For UDG treatment, 0.5 × UDG buffer and 0.5 U of uracil-DNA glycosylase (New England Biolabs) were added to the PCR master mix. The same PCR conditions were used except an addition of initial incubation at 37°C for 30 min before the activation of HotStarTaq polymerase.

A PIK3CA E549D mutation detected at a low frequency (4%) in the Core 2 of Tumor 2 by AmpliSeq was analysed by limited copy number (LCN)-HRM [29]. The reaction mixture and cycling conditions were those used for the FGFR3 HRM assay with UDG treatment, except with an annealing temperature of 60°C and 100 pg of template were used. The Core 2 sample was tested in 100 replicates, but no variants were identified. The primer sequences for PIK3CA exon9 analysis were F 5′- AAGAACAGCTCAAAGCAATTTCTACAC-3′ and R 5′-AATCTCCATTTTAGCACTTACCTGTGAC-3′.

### Functional assays

Single cell colony formation assays involved seeding single cells in 96 well plates and counting the wells with colonies of greater than 50 cells after four weeks.

Soft agar colony formation assays were performed in 24-well plates, with 2000 cells cultured in 500 μL of RF10 in 0.9% agarose, on a base layer 1.4% agarose in RF10. 500 μL of RF10 was layered over top of the agarose, and plates were incubated for four weeks at 37°C. Colonies were visualized and counted follow staining with MTT (3-(4,5-Dimethylthiazol-2-yl)-2,5-diphenyltetrazolium bromide; Sigma Aldrich) for 60 minutes.

Paclitaxel and 5FU (Sigma Aldrich) were resuspended in DMSO and sterile water respectively. Drug treatments were applied to 7500 cells/well in 100 μL RF10 in 96-well plates, with final concentrations of 20 nM paclitaxel and 200 μM 5FU. After 72 hours the amount of viable cells remaining was quantified using the CellTiter AQueous One Solution Cell Proliferation Assay (Promega). Values from drug treatments were compared to vehicle-only controls. A paclitaxel-resistant derivative of LM-MEL-62 was created by exposing the parental cell line to drug for 72 hours, then allowing the cells to recover to confluence before re-treating. Three rounds were performed, with doses doubled each time, with a final treatment of 40 nM. All functional assays were performed on cell line clones before they had reached ten passages in culture.

### Quantitative PCR (QPCR)

cDNA was produced from RNA using the High Capacity cDNA Reverse Transcription Kit (Applied Biosystems). QPCR was performed using the QuantiFast SYBR Green PCR Kit (Qiagen), with reactions run on a Stratagene MX3005P thermocycler. Expression levels were normalized to beta-actin. Primer sequences were as follows: *IFI6* F 5′- AGCAGCAGGTAGCACAAGAA-3′ and R 5′- GGGCTGAAGATTGCTTCTCTT -3′; *IFI27* F 5′-GCCACAACTCCTCCAATCAC-3′ and R 5′- ATCAGCAGTGACCAGTGTGG -3′; *MX1* F 5′- GATGATCAAAGGGATGTGGC -3′ and R 5′- AGCTCGGCAACAGACTCTTC -3′.

## Competing interests

The authors declare that they have no competing interest.

## Authors’ contributions

MA performed molecular and cell biology experiments, analysed the microarray and amplicon sequencing datasets, participated in study design and coordination, and wrote the manuscript. CH performed cell biology experiments. PHL performed cell biology experiments. HD performed the high-resolution melt validation experiments. OLC participated in study design, coordination, and provision of materials. IDD participated in study design and coordination, and helped draft the manuscript. JC participated in study design and coordination, and helped draft the manuscript. AB conceived of the study, performed cell culture experiments, participated in study design and coordination, and helped draft the manuscript. All authors read and approved of the final manuscript.

## Pre-publication history

The pre-publication history for this paper can be accessed here:

http://www.biomedcentral.com/1755-8794/6/40/prepub

## Supplementary Material

Additional file 1: Figure S1 Shows H&E staining of sections from FFPE blocks of Tumor 1. Inserts are H&E stains from the bottom of the core fragment used for DNA isolation. Scale bar next to whole section represents 1 mm, bars next to cores represent 100 μm. The section taken from below Core 1 in Block 1–4 shows few tumor cells as the tissue in this area was thin and the core passed through the remaining tissue. It is worth noting that during the process of cutting sections for microscopy several micrometers of thickness from the block were shaved off and discarded before usable sections can be obtained. Thus the post coring images are not exact representations of the tissue that was immediately underneath that core fragments which were used for DNA extraction. Click here for file

Additional file 2: Figure S2 Shows copy number heterogeneity of Chromosome 17 in different regions of Tumor 1. Segmentation results for all eight cores are shown in the top panel; amplification in red, deletions in blue. The plots below show results for Block 1-1 Core 2 and Block 1-1 Core 3 in greater detail. Regions defined by segmentation are highlighted by solid red and blue bars as above. Small dots represent the copy number values of individual array probes, while larger dots represent smoothed data resulting from averaging results from 30 adjacent probes. Click here for file

Additional file 3 Supplementary Results. Click here for file

Additional file 4: Table S1 Is an Excel file containing the results from the Ion Ampliseq Cancer Panel variant analysis performed on DNA from cores of Tumors 1, 2 and 3. Only one transcript was listed where the amino acid change was equivalent across isoforms. Click here for file

Additional file 5: Figure S3 Shows the identification and validation of sequence mutations in cancer genes in different regions of metastatic melanoma tissue samples. A) Fluorescence intensities for Oncoscan probes specific for cancer mutations in cores from Tumor 1. B) Examples of chromatograms from capillary sequencing of regions of *NRAS* and *MSH2* predicted to be mutated in Tumor 1 based on Oncoscan probe intensities. C) Normalized HRM plots for PCR products covering *FGFR3* exon 6 before and after UDG treatment. Click here for file

Additional file 6: Figure S4 Shows H&E stainings of Tumors 2 and 3. Only one block was available for Tumor 2. Tumor 3 Blocks 1–1 through 1–4 are from a spleen metastasis, Block 2–4 is a hepatic artery lymph node metastasis removed concurrently from the same patient. White numerals indicate regions where cores were removed. Click here for file

Additional file 7: Table S2 Is an Excel file containing GSEA results from comparing the clones in each cluster shown in Figure 8A. Click here for file
