# Supplementary figures and images for: Intratumoral genetic heterogeneity in metastatic melanoma is accompanied by variation in malignant behaviors
Source: BMC Med Genomics. 2013 Oct 11;6:40. doi: 10.1186/1755-8794-6-40 (PMC3852494; doi:10.1186/1755-8794-6-40)

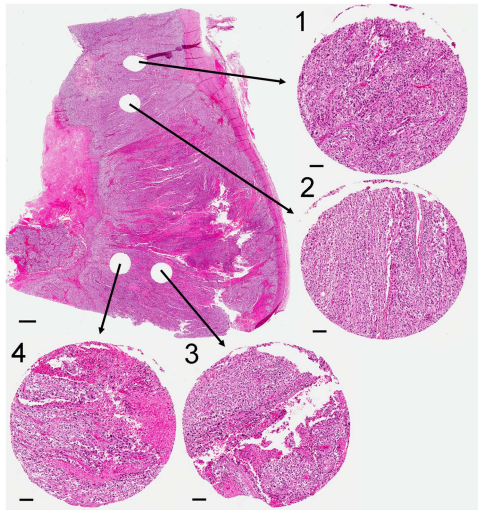

Block 1-1

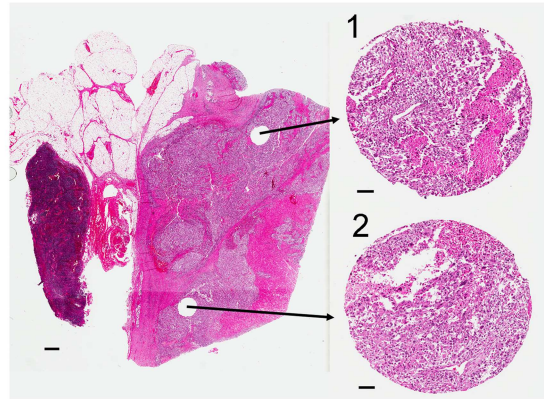

Block 1-2

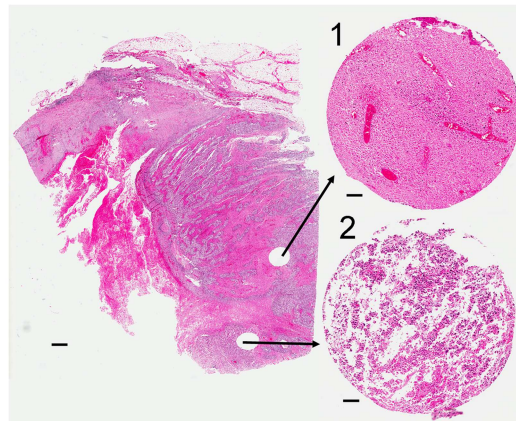

Block 1-4

Supplement: Additional file 1: Figure S1 — Shows H&E staining of sections from FFPE blocks of Tumor 1. Inserts are H&E stains from the bottom of the core fragment used for DNA isolation. Scale bar next to whole section represents 1 mm, bars next to cores represent 100 μm. The section taken from below Core 1 in Block 1–4 shows few tumor cells as the tissue in this area was thin and the core passed through the remaining tissue. It is worth noting that during the process of cutting sections for microscopy several micrometers of thickness from the block were shaved off and discarded before usable sections can be obtained. Thus the post coring images are not exact representations of the tissue that was immediately underneath that core fragments which were used for DNA extraction. [file 1755-8794-6-40-S1.pdf]

Amplification ■ Deletion ■

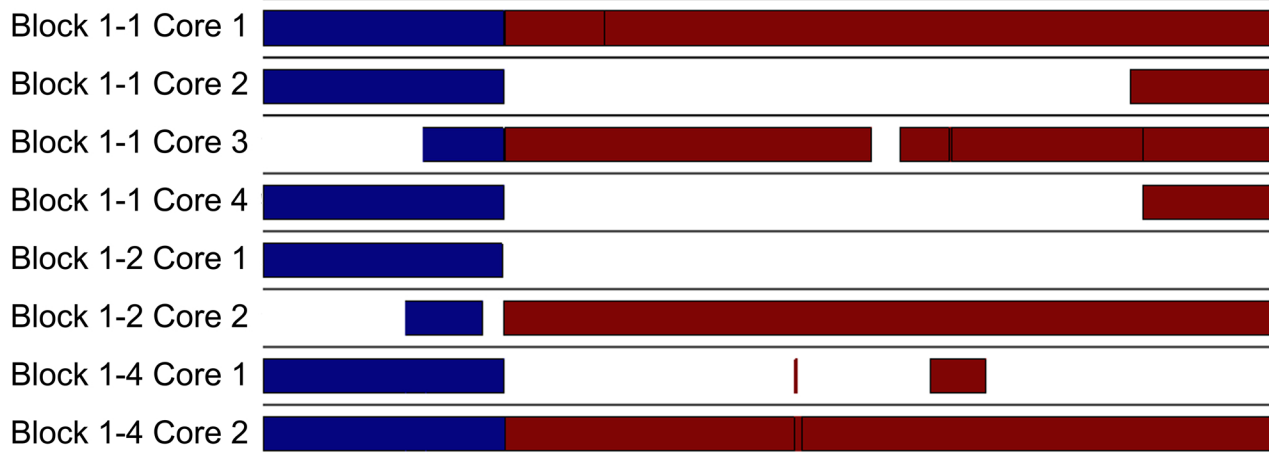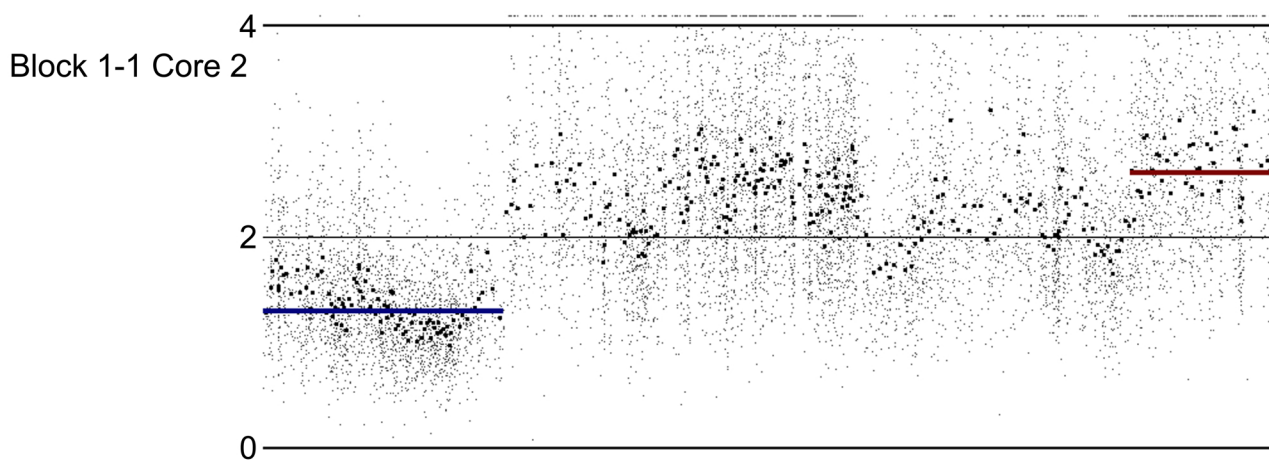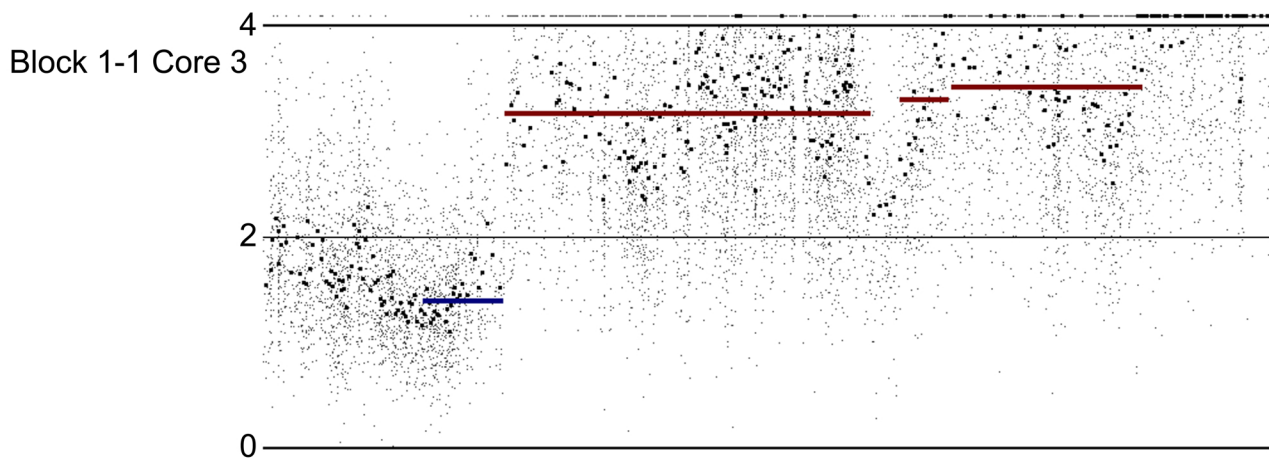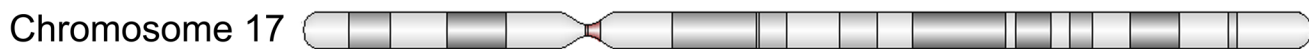

Supplement: Additional file 2: Figure S2 — Shows copy number heterogeneity of Chromosome 17 in different regions of Tumor 1. Segmentation results for all eight cores are shown in the top panel; amplification in red, deletions in blue. The plots below show results for Block 1-1 Core 2 and Block 1-1 Core 3 in greater detail. Regions defined by segmentation are highlighted by solid red and blue bars as above. Small dots represent the copy number values of individual array probes, while larger dots represent smoothed data resulting from averaging results from 30 adjacent probes. [file 1755-8794-6-40-S2.pdf]

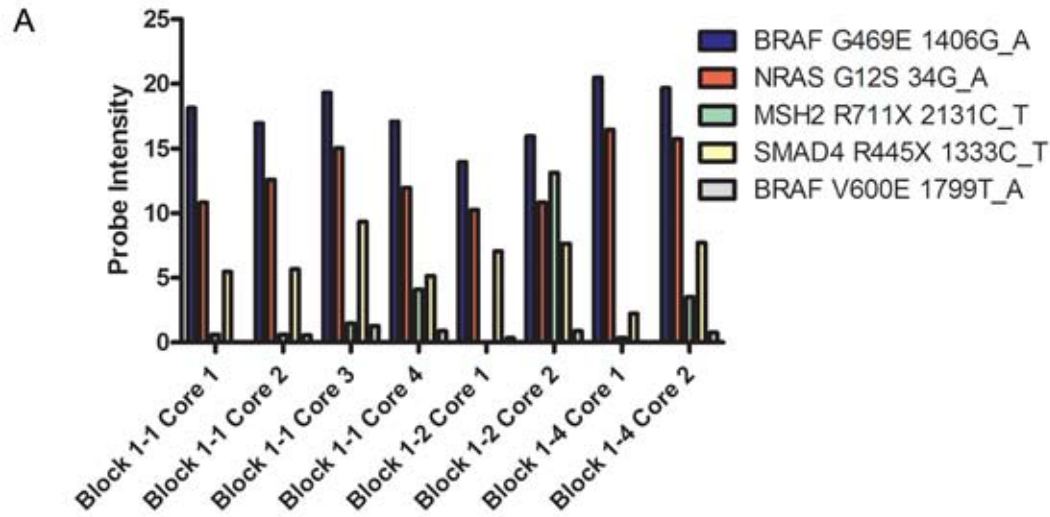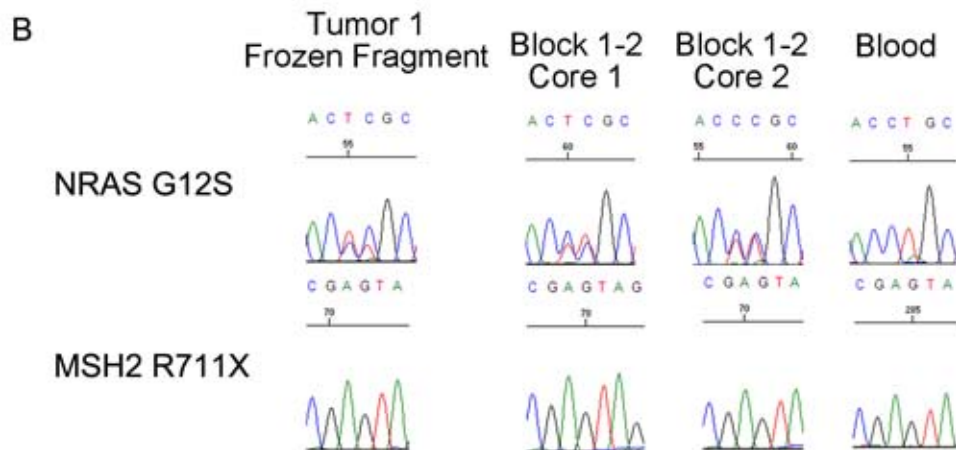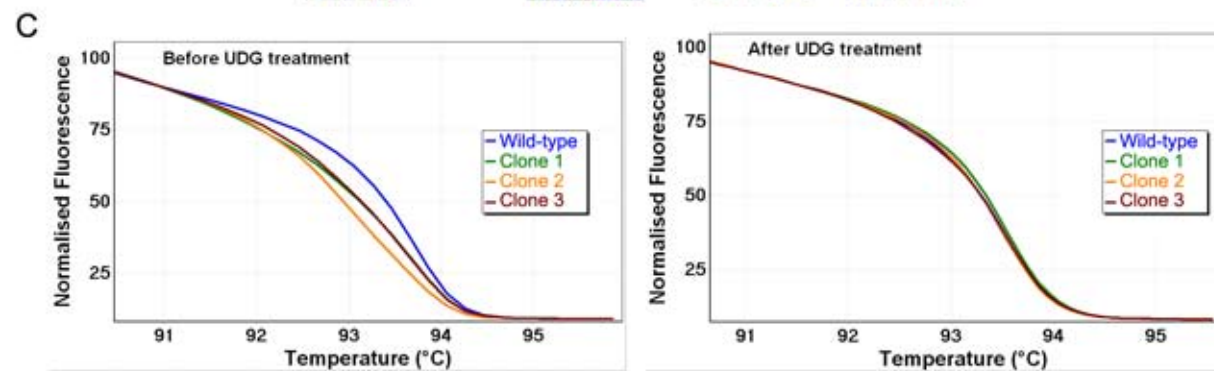

Supplement: Additional file 5: Figure S3 — Shows the identification and validation of sequence mutations in cancer genes in different regions of metastatic melanoma tissue samples. A) Fluorescence intensities for Oncoscan probes specific for cancer mutations in cores from Tumor 1. B) Examples of chromatograms from capillary sequencing of regions of NRAS and MSH2 predicted to be mutated in Tumor 1 based on Oncoscan probe intensities. C) Normalized HRM plots for PCR products covering FGFR3 exon 6 before and after UDG treatment. [file 1755-8794-6-40-S5.pdf]

Tumor 2

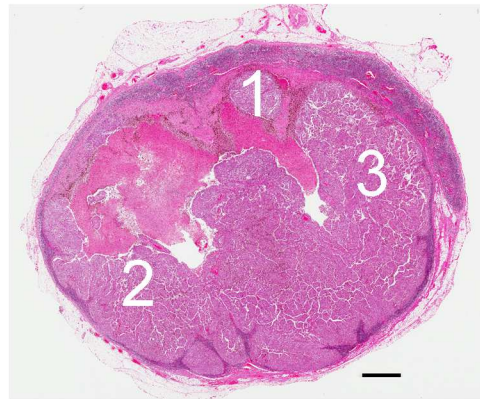

Tumor 3

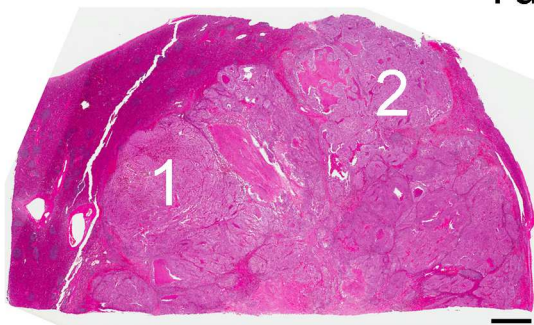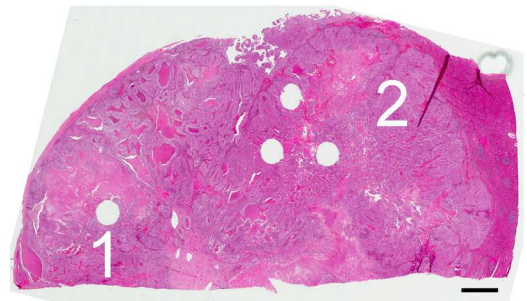

Block 1-1

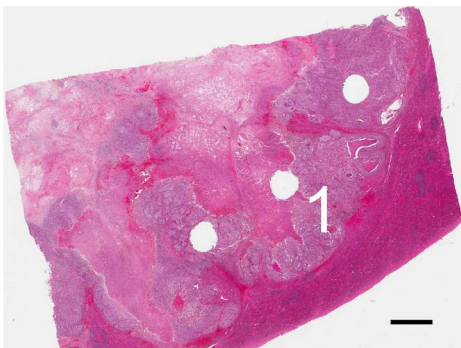

Block 1-2

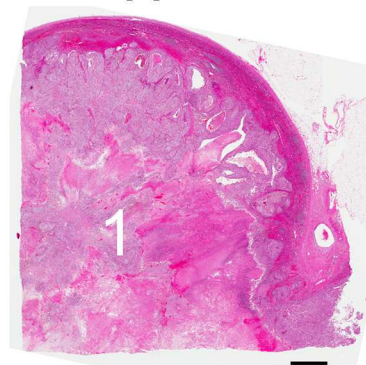

Block 1-3

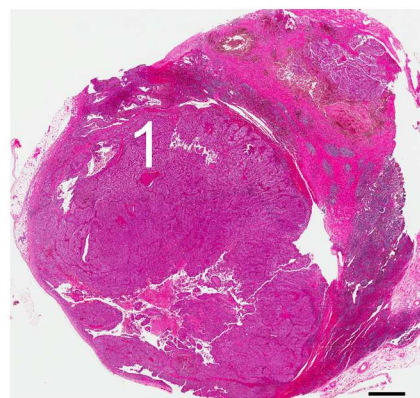

Block 1-4

Block 2-4

Supplement: Additional file 6: Figure S4 — Shows H&E stainings of Tumors 2 and 3. Only one block was available for Tumor 2. Tumor 3 Blocks 1–1 through 1–4 are from a spleen metastasis, Block 2–4 is a hepatic artery lymph node metastasis removed concurrently from the same patient. White numerals indicate regions where cores were removed. [file 1755-8794-6-40-S6.pdf]
