# Supplementary material for: Intratumoral genetic heterogeneity in metastatic melanoma is accompanied by variation in malignant behaviors
Source: BMC Med Genomics. 2013 Oct 11;6:40. doi: 10.1186/1755-8794-6-40 (PMC3852494; doi:10.1186/1755-8794-6-40)
Supplement: Additional file 3 — Supplementary Results. [file 1755-8794-6-40-S3.docx]

**Additional file 5**

**Supplementary Results**

**Mutation profiling**

The Oncoscan 2.0 platform includes probes that test for 541 individual mutations in 62 well known cancer genes, with the presence of a mutation indicated by high probe intensity. In the dataset derived from the eight cores from Tumor 1, the maximum normalized probe intensity was 20.5, and the median was 0.22. Affymetrix recommends an intensity of nine as a cut off to select mutations for validation by sequencing. Probes for mutations in *BRAF* (G469E) and *NRAS* (G12S) displayed consistently high intensities in all eight cores, and probes interrogating *MSH2* (R711X) and *SMAD4* (R445X) demonstrated high intensity in DNA from some regions but not others (Supplementary Figure S3A). We attempted to validate these mutations using standard capillary sequencing (examples shown in Supplementary Figure S3B). The NRAS and BRAF mutations were found in all regions of Tumor 1, as well as in DNA from an independent fresh frozen tumor fragment, but not in DNA from the patient’s blood. The *MSH2* and *SMAD4* mutations could not be identified in the DNA from any region of Tumor 1.

As capillary sequencing cannot detect mutations present at low frequency in DNA, we used a next-generation sequencing approach to attempt to validate the heterogeneous presence of sequence mutations in cancer genes. The Ion Ampliseq Cancer Panel (Invitrogen) covers 46 oncogenes and tumor suppressors, the majority of which were also present on the Oncoscan platform. Five of the eight DNA samples from Tumor 1 previously analysed using the Oncoscan platform were sequenced, as were multiple regions of tumors from two other patients (Tumors 2 and 3). H&E stains for these additional tumors are shown in Supplementary Figure S4, and full results tables from the variant analysis are available in Supplementary Table S1. The Ampliseq results confirmed the presence of *BRAF* (G469E) and *NRAS* (G12S) mutations in all regions of Tumor 1, and also identified a mutation in *TP53* (S127F) in all regions. All regions of Tumor 2 were identified as containing a mutation in *ATM* predicted to effect protein function, as well as an activating mutation in *NRAS* (Q61R). All regions of Tumor 3 contained a *BRAF* V600K mutation.

*MSH2* was not covered by the Ampliseq panel. *SMAD4* was included; however the R445X mutation was not identified in any regions of Tumor 1. The Ampliseq panel identified several variants in each tumor that appeared to be present in some regions but not others. All of these variants were present at low frequency variants (between 3-25% of reads) and were sequenced at low relative coverage. Four different FGFR3 variants were detected in the three cores of Tumor 2 (2 variants in Core 1, 1 variant in Cores 2 and 3). All FGFR3 variants were either C>T or G>A changes, and none were found in more than one core. Transitional C:G>T:A artifacts can be generated from uracil lesions in FFPE DNA as a result of cytosine deamination (Do and Dobrovic 2012). Therefore DNA from each core was tested using high resolution melting before and after uracil-DNA glycosylase (UDG) treatment, as the treatment of FFPE DNA with UDG specifically reduces C:G>T:A artefacts arising from uracil lesions. Based on the elimination of the melting temperature shift by UDG treatment, these variants appeared to formalin fixed tissue artifacts (Supplementary Figure S3C). A PIK3CA E549D mutation apparently detected at a low frequency (4%) in Core 2 of Tumor 2 was analysed by limited copy number (LCN)-HRM (Do and Dobrovic 2009); however, in 100 replicate reactions no mutation was detected (data not shown). We therefore concluded that the variably present, low frequency mutations identified by the Oncoscan platform and the Ampliseq panel were the results of formalin fixation of the tissue.

Based on these results we concluded that melanoma metastases are homogeneous for driver mutations that likely represent early events during tumor progression, such as those affecting MAPK pathway components. Meanwhile despite using two platforms designed to be used with FFPE tissues, we detected many false positive mutations likely related to fixation that gave the appearance of heterogeneity.

**Supplementary References**

Do, H. and A. Dobrovic (2009). "Limited copy number-high resolution melting (LCN-HRM) enables the detection and identification by sequencing of low level mutations in cancer biopsies." Mol Cancer **8**: 82.

Do, H. and A. Dobrovic (2012). "Dramatic reduction of sequence artefacts from DNA isolated from formalin-fixed cancer biopsies by treatment with uracil- DNA glycosylase." Oncotarget **3**(5): 546-558.
